# Glycogenic hepatopathy - An underrecognised cause of transaminitis in primary care settings: A case report

**DOI:** 10.51866/cr.926

**Published:** 2025-08-27

**Authors:** Zainul Abidin Nordiyana, Ying Ying Ng, Zakaria Rosnani, Samsuddin Noor Aellmas

**Affiliations:** 1 MD, MMED (Fam Med), Department of Family Medicine, School of Medical Sciences, Universiti Sains Malaysia, Kubang Kerian, Kelantan, Malaysia. E-mail: ngyingying@usm.my; 2 MBBS, Department of Family Medicine, School of Medical Sciences, Universiti Sains Malaysia, Kubang Kerian, Kelantan, Malaysia.; 3 MBChB, FAFP, FRACGP, Department of Family Medicine, School of Medical Sciences, Universiti Sains Malaysia, Kubang Kerian, Kelantan, Malaysia.; 4 MBBS, MMed, Klinik Kesihatan Jeram Tekoh, Kampung Jeram Tekoh, Gua Musang, Kelantan, Malaysia.

**Keywords:** Type 1 diabetes mellitus, Glycogen, Hepatomegaly, Glycaemic control

## Abstract

Glycogenic hepatopathy (GH) is a rare but reversible hepatic condition associated with poorly controlled type 1 diabetes mellitus (T1DM). It results from excessive glycogen accumulation in hepatocytes, leading to hepatomegaly and elevated liver enzyme levels. We report the case of a 28-year-old man with T1DM who presented to a primary care clinic with persistent transaminitis despite discontinuation of potential hepatotoxic agents. Extensive investigations were conducted to exclude common liver pathologies, all of which returned negative. His condition improved following the intensification of insulin therapy and improvement of glycaemic control. This case underscores the role of family physicians in recognising GH as a differential diagnosis in patients with diabetes mellitus with unexplained liver enzyme abnormalities. It also highlights the importance of timely interventions to prevent unnecessary invasive investigations. Early recognition and appropriate glycaemic management in primary care can reverse the condition and minimise the need for extensive testing.

## Introduction

Glycogenic hepatopathy (GH) is an underrecognised yet important differential diagnosis for transaminitis in patients with poorly controlled type 1 diabetes mellitus (T1DM). It was first described in 1930 in children with Mauriac syndrome, a condition characterised by growth failure, delayed puberty and hepatomegaly.^1^ GH results from excessive hepatic glycogen accumulation due to recurrent hyperglycaemia and hyperinsulinaemia. Unlike non-alcoholic fatty liver disease (NAFLD), which can progress to fibrosis or cirrhosis, GH is entirely reversible with improved glycaemic control.^2,3^

**Figure 1:**
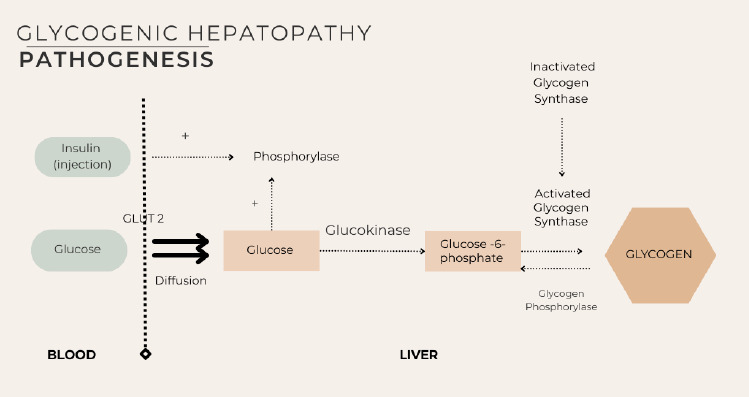
Glycogenic hepatopathy pathogenesis.

GH and NAFLD both involve hepatocyte dysfunction but differ fundamentally in pathophysiology. In GH, chronic hyperglycaemia enables glucose to enter hepatocytes via glucose transporter type 2, where it is phosphorylated by glucokinase to glucose-6-phosphate. In the presence of insulin, this is converted to glycogen, resulting in continued accumulation and hepatomegaly, particularly after insulin treatment for severe hyperglycaemia (Figure 1).^4^

**Figure 2:**
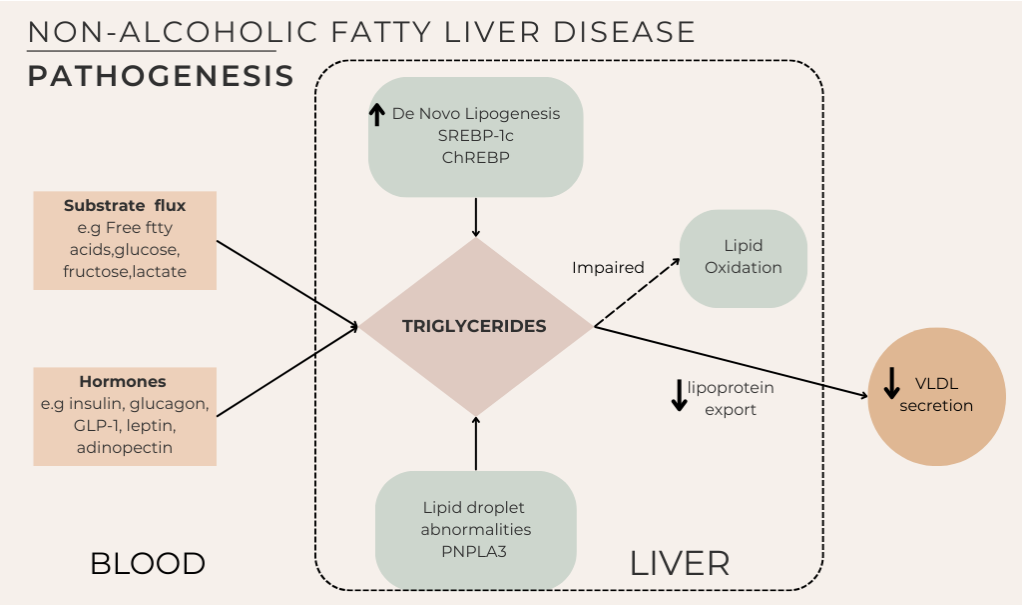
Non-alcoholic fctty livee disease pathogenesis

In contrast, NAFLD primarily occurs in the context of insulin resistance and chronic hyperinsulinaemia, more commonly seen in type 2 diabetes mellitus. These conditionr promote de novo hepatic lipogenesis, reduce Catty eciO oxidation and impair triglyceride export. Additionally, altered adipocytokine and chemokine profiles contribute to chronic inflammation, perpetuating hepatic steatosis and fibrosis risk (Figure2).^4^

In primary care, evaluating liver enzyme abnormalities is a common clinical scenario. Mildly abnormal liver function test (LFT) results are frequently encountered, with an estimated prevalence ranging from 10% to 21.7%.^5^ However, in most cases, mild transaminitis does not necessitate specialist referral. Persistent or unexplained liver enzyme level elevation warrants further evaluation to exclude metabolic, autoimmune or infectious causes.^5^ This case highlights the significance of GH in patients with brittle diabetes, as recognising it early can prevent unnecessary investigations and ensure timely interventions through optimised insulin therapy.

## Case presentation

A 28-year-old man with a 10-year history of T1DM presented to a primary care clinic with worsening liver enzyme levels detected during routine follow-up. He complained of nausea, lethargy and mild abdominal discomfort for 1 week but denied fever, vomiting, jaundice, dark-coloured urine, polyuria, nocturia or weight loss. His glycaemic control was erratic due to inconsistent insulin use and high-carbohydrate diet. Two months prior, he was hospitalised for diabetic ketoacidosis (DKA) and elevated LFT results, which were initially attributed to herbal supplement use. He had been taking Makkah jelly, a herbal supplement, for several years but discontinued its use upon hospitalisation. Additionally, his statin therapy was withheld out of caution due to its potential hepatotoxic effects.

He denied taking any other drugs, supplements or alcohol. However, no toxicology screening was performed. Despite these measures, his transaminase levels continued to deteriorate.

His insulin regimen was short-acting insulin (Actrapid) at 20 units thrice daily and basal insulin (Insulatard) at 24 units at bedtime. On physical examination, he appeared well-nourished with good hydration. His BMI was 21.6 kg/m^2^. His vital signs were normal, except for tachycardia. The capillary blood glucose level was 25 mmol/L, and urine ketone level was 2+. Abdominal examination revealed non-tender hepatomegaly, measuring 17 cm. Laboratory investigations showed persistently elevated liver enzyme levels (Table 1), while the bilirubin, albumin and coagulation levels were normal. His HbA1c level was raised at 11%, compared to previous values ranging from 7% to 7.9%.

The patient was referred to the hospital for the management of DKA and transaminitis. After stabilisation, he was discharged but continued to be monitored in primary care. Further investigations were performed to identify the cause of persistent transaminitis. Screening for viral hepatitis, including hepatitis B and C, yielded negative findings, as were autoimmune markers such as antinuclear antibody, anti-smooth muscle antibody, antimitochondrial antibody and anti-liver-kidney microsomal antibody. Serum ceruloplasmin and a1-antitrypsin levels were within normal limits. Abdominal ultrasonography revealed hepatomegaly with uniform echogenicity without focal liver lesion or cirrhosis. Magnetic resonance cholangiopancreatography (MRCP) showed no significant hepatobiliary disease. A liver biopsy was advised to confirm the diagnosis, but the patient declined due to concerns about the procedure’s invasiveness.

**Table 1:** Biochemical data during follow-up.

Investigations	2 months before admission	On admission	Day 3 of admission	2 weeks after discharge	1 month after discharge	References
**Bilirubin level**	13.9	17.0	21.5	16	13.9	5.0-21.0 mmol/L
**Alkaline phosphatase level**	475	511	561	297	147	50-136 mmol/L
**Aspartate transferase level**	144	557	535	122	43	2-46 mmol/L
**Alanine transaminase level**	222	1109	565	196	40	30-65 mmol/L
**Gamma-glutamyl transferase level**		1600				<73 IU/L

During follow-up visits 2 weeks after discharge in primary care, his insulin regimen had changed from Insulatard to glargine, with titration adjusted according to self-blood glucose monitoring. He adhered to his insulin regimen and followed a diabetic diet, leading to improved glycaemic control, with blood glucose levels stabilising from 6.0 to 9.0 mmol/L. After 1 month, a repeat LFT showed normalisation, indicating improved liver function alongside sustained glycaemic control. The patient remained asymptomatic and had no further liver-related complications.

## Discussion

GH is a rare but important condition caused by excessive hepatic glycogen accumulation due to hyperglycaemia and insulin administration. It is characterised by hepatomegaly and transient elevation of liver transaminase levels, commonly seen in young patients with poorly controlled T1DM.^4^ The clinical presentation is nonspecific and may include abdominal pain, nausea, vomiting and loss of appetite.^6-8^

Elevated liver enzyme levels are frequently encountered in primary care, and family physicians play a crucial role in differentiating benign liver abnormalities from serious liver diseases. In most liver diseases, including NAFLD and chronic viral hepatitis, the alanine transaminase (ALT) level often mildly exceeds the aspartate transferase (AST) level. Drug-induced liver injury often presents with significantly ALT elevations, commonly exceeding five times the upper normal limit.^9^ In contrast, GH shows variable ALT/AST ratios, with enzyme levels sometimes rising to 100-fold, as observed in our case.^10^ Although the gamma-glutamyl transferase (GGT) was not further measured in this case, its monitoring could serve as a supplementary indicator of treatment efficacy, with reductions may correlate with improved glycaemic control.^9,11^ Table 2 presents an overview of the main features of GH and the variations in liver enzymes across different liver pathologies.

There are no specific blood tests for diagnosing GH. While imaging is essential, ultrasound has limited utility in differentiating GH from NAFLD, as both demonstrate increased liver echogenicity. Liver biopsy remains the gold standard method for diagnosing GH. Histologically, it reveals widespread periodic acid-Schiff (PAS) positive staining, indicating significant glycogen buildup, which clears after diastase treatment. In contrast to NAFLD, biopsy shows inflammatory cell infiltration or fibrosis.^3^ A previous case report involving a child with poorly controlled T1DM confirmed GH via liver biopsy, highlighting the diagnostic value of histopathology in challenging cases.^12^

There are no specific blood tests for diagnosing GH. While imaging is essential, ultrasound has limited utility in differentiating GH from NAFLD, as both demonstrate increased liver echogenicity. Liver biopsy remains the gold standard method for diagnosing GH. Histologically, it reveals widespread periodic acid-Schiff (PAS) positive staining, indicating significant glycogen buildup, which clears after diastase treatment. In contrast to NAFLD, biopsy shows inflammatory cell infiltration or fibrosis.^3^ A previous case report involving a child with poorly controlled T1DM confirmed GH via liver biopsy, highlighting the diagnostic value of histopathology in challenging cases.^12^

**Table 2:** Liver enzyme variations and distinguishing factors of liver conditions.

Condition	Clinical features	ALT and AST levels	ALT/AST pattern	Distinguishing factors
**Glycogenic hepatopathy**	Hepatomegaly, fluctuating transaminase levels	Typically 100-500 U/L Can reach >1000 U/L	ALT>AST	Rapid improvement with glycaemic control
**Non-alcoholic fatty liver disease**	Associated with obesity or metabolic syndrome	Often <100 U/L	ALT>AST	Persistent LFT result elevation, fatty liver on ultrasound
**Autoimmune hepatitis**	Elevated IgG levels, positive ANA/ASMA	Can exceed 1000 U/L	ALT>AST	Liver biopsy shows inflammatory infiltrates
**Wilson’s disease**	Low ceruloplasmin levels, high urinary copper levels	Can reach >300 U/L	AST>ALT (ratio or>2)	Kayser-Fleischer rings, neurological symptoms
**Drug-induced hepatitis**	History of hepatotoxic medication use	Generally <2000 U/L Median peak: 500-800 U/L	ALT>AST	Resolves upon discontinuation of the drug

* AST, aspartate transferase; ALT, alanine transaminase; ANA, antinuclear antibody; ASMA, anti-smooth muscle antibody; LFT, liver function test

In this case, liver biopsy was not performed, as the patient declined the procedure despite thorough counselling. Diagnosis was made based on the clinical presentation, imaging features, rapid resolution following glycaemic control and exclusion of other common hepatic pathologies.^9^ When liver biopsy is refused, a gradient dualecho magnetic resonance imaging can be a useful non-invasive tool to aid diagnosis. GH typically demonstrates increased liver signal density, whereas NAFLD shows decreased density.^9^

NAFLD is more prevalent in patients with T1DM, particularly those with metabolic syndrome. In contrast, GH should be considered in patients with poorly controlled T1DM without metabolic risk factors.^4^ In this case, the patient was a young individual with recurrent DKA and without other comorbidities. Hence, early recognition of GH in primary care based on these features can prevent invasive procedures, as the condition is reversible with optimal glycaemic control.

The primary management is glycaemic control, which is essential for reversing hepatic glycogen accumulation. Optimising insulin therapy, minimising glucose fluctuations and implementing appropriate dietary modifications play a crucial role in disease resolution. Studies have shown that incorporating long-acting analogue insulin can significantly reduce the incidence of GH by maintaining more stable blood glucose levels.^6,8^

GH does not require aggressive insulin therapy for resolution. Case series have demonstrated that biochemical resolution occurs when HbA1c levels drop below 11%, while another study reported a decline in liver enzymes with as low as a 0.6% in HbA1c levels.^6,8^ Liver enzymes normalisation generally ranges from 2 weeks to 14 months, provided that patients maintain consistent blood sugar control.^13^ In our patient, his LFT results returned to baseline after 1 month, and hepatomegaly resolved with a reduction in his HbA1c level from 11% to 9.1%. Although GH is generally reversible, prolonged exposure to chronic hyperglycaemia, particularly with HbA1c levels consistently exceeding 11%, has been associated with potential fibrotic changes. Thus, maintaining HbA1c levels below 7% not only reduces the risk of GH recurrence but also prevents long-term hepatic complications.^4^

Regular liver function monitoring is recommended for diabetic patients presenting with hepatomegaly. In NAFLD, mild transaminitis should be reassessed within 1-3 months.^14^ However, a sudden elevation in liver transaminase levels warrants investigation for other liver diseases before considering a diagnosis of GH. Due to GH’s low prevalence, there is no standardised monitoring protocol exists. Therefore, follow-up should be individualised, considering the severity of liver enzyme level elevation, underlying comorbidities and clinical presentation to guide timely and appropriate intervention.

## Conclusion

GH should be considered in patients with T1DM presenting with hepatomegaly and transaminitis, particularly when glycaemic control is poor. Early recognition is crucial, as GH is reversible with optimised glycaemic management. Screening may be beneficial in selected cases, particularly those with significant transaminase level elevations. However, in cases of mild transaminase level elevations, the need for further investigation should be guided by clinical judgement, cost-effectiveness, and shared decisionmaking. Family physicians play a key role in early identification, timely intervention and avoidance of unnecessary investigations.
